# Comparison among three kinds of assessment for the Mild Behavioral Impairment Checklist (MBI‐C)

**DOI:** 10.1002/alz.093564

**Published:** 2025-01-03

**Authors:** Tzung‐Jeng Hwang, Cho‐Hsiang Yang, Yi‐Ting Lin

**Affiliations:** ^1^ National Taiwan University Hospital, College of Medicine, National Taiwan University, Taipei Taiwan; ^2^ National Taiwan University Hospital‐Bei Hu Branch, Taipei, Taiwan Taiwan; ^3^ National Taiwan University Hospital, Taipei City Taiwan

## Abstract

**Background:**

Mild Behavioral Impairment (MBI) is a construct that describes the emergence of sustainable and at least mildly impactful neuropsychiatric symptoms (NPS) after age 50. The Mild Behavioral Impairment Checklist (MBI‐C) is a provisional tool used to measure the NPS of MBI. However, who should provide the information for the assessment of the NPS remained unclear.

**Method:**

We recruited 123 MBI participant‐informant dyads from a psychiatric outpatient clinic of a university medical center. The MBI‐C was administered to both the participant (MBI‐self) and informant (MBI‐I). Then, a researcher (psychologist) compared the two results, clarified the differences, and made the final assessment (MBI‐R).

**Result:**

There were 49 with MBI only and 74 with MBI plus MCI. Among the 123 MBI subjects, the mean MBI‐C total score was 6.06 (SD 6.7), 5.27 (SD 7.67), and 6.34 (SD 6.4) for MBI‐S, MBI‐I, and MBI‐R. There was no significant difference among the three. However, for the motivation domain, the MBI‐S and MBI‐R scoring was significantly higher than the MBI‐I scoring (p < 0.001). For the affect domain, the MBI‐R scoring was significantly higher than the MBI‐I scoring (p = 0.019). For the social domain, the MBI‐I scoring was significantly higher than the MBI‐S scoring (p = 0.012). There were no significant differences among the 3 scorings for the impulse or psychosis domains. When we examined each item in the 5 domains, the general tendency was consistent with the findings mentioned above, except that there were 3 items in the impulse domain showing a significant difference among the 3 scorings (MBI‐I, MBI‐R > MBI‐S).

**Conclusion:**

Our findings showed there might be differences among the 3 kinds of scorings for the 5 MBI domains. If the MBI‐R scoring was treated as the gold standard, it was close to the MBI‐S scoring but higher than the MBI‐I scoring for the motivation and affect domain. For the social domain, the MBI‐R scoring was close to the MBI‐I but higher than the MBI‐S.

